# Accuracy in Analysis: The Role of Standard Reference Materials

**DOI:** 10.6028/jres.093.020

**Published:** 1988-06-01

**Authors:** Stanley D. Rasberry

**Affiliations:** Office of Standard Reference Materials, National Bureau of Standards, Gaithersburg, MD 20899

## 1. Introduction

What one question haunts the best of analytical chemists when their day’s work is done? Four of the main questions that arise regarding any analytical method are:

—Is it sensitive enough for the level of detection required?
—Is it free of interferences for the desired analyte?
—Is it precise, so that the results are reproducible?
—Is it accurate, so that the results approach true values?

Probably it is the last of these questions that brings the greatest difficulty and the most soul searching to the analyst. If this were not the case, why are there so many cars in chemistry building parking lots on weekends and holidays? Why are analysts often reluctant to report results without “just one more retest”?

This paper will attempt to pick apart some of these questions. While it may answer none of them conclusively, it is aimed at demonstrating the role of Standard Reference Materials (SRMs) in the analyst’s pursuit of accuracy. In the case of trace analysis near the detection limit of state-of-the-art methods, certifiers of reference materials face a very special problem: certified error limits often seem unacceptably high when considered on a relative basis. This paper will include a brief discussion of this problem.

## 2. Role of Standard Reference Materials (SRMs)

Simply stated, the National Bureau of Standards (NBS) produces SRMs to help people bring quality assurance to their measurements. In some cases instruments require SRMs for calibration. In most cases measurement accuracy should be validated by use of a certified reference material.

In the mining and manufacturing industries, at least four major stages of activity require measurements and thus some form of measurement quality assurance:

—Raw Materials.
—Finished Materials.
—Subcomponents.
—Products.

At the first two stages, the measurements are most likely to be chemical analyses. At later production stages, measurements are more apt to be physical or engineering tests. Increasingly, SRMs are also being produced for use in these later stages of production.

Today, the U.S. economy has shifted from manufacturing to an emphasis on the service sector. Chemical analysis plays an important role in this sector, too. Here we often find measurement quality assurance needs for analyses in such service areas as: environmental, clinical, geological, and energy. SRMs are underutilized by the service sector and could be very helpful to future improvements in quality assurance of measurements.

A third community that has a heavy focus on measurements is the academic and R&D community. Here is where we get most of our new concepts for instrumentation and measurement methods. When new developments are tried out in the lab and reported in the literature, SRMs have a critical role in helping the researchers assess the accuracy of what they have wrought.

## 3. Concern About Accuracy

In certifying SRMs, the goal at the NBS is to give a true value at a stated level of accuracy for each property certified [1]. Notice the word accuracy rather than precision. The concept of accuracy is focused on arriving at the “true” or “actual” value of a property. Real world materials are usually not completely uniform, so the true value may differ for different samples taken from the material. Provided the differences are small, they may be covered in an accuracy statement representative of the entire lot. Large differences require rejection of the lot or individual certification of each sample.

Conversely, precision gives no indication of how closely a set of measurements approaches the true value. The concept of precision is focused on how tightly clustered a set of measurements is. Said another way, one can have a set of measurements which is extremely precise, but also extremely wrong. Precision may be viewed as a necessary but not sufficient precursor of accuracy.

I have tried to establish an estimate of accuracy that a wide range of chemists and methods realize at trace levels. To do this, I have used a literature survey [2] which has reviewed more than 100 papers citing results of analysis of SRM 1571, Orchard Leaves. In table 1 are summarized the results found for two elements (iron and aluminum) which are present at about 300 μg/g. Note that iron is certified by NBS, while aluminum is not. Similarly, one certified element (strontium) and one element not certified (titanium) in SRM 1571 are shown and are at about one order of magnitude lower concentration. A more detailed view of the analytical accuracy is given in the next two tables.

Table 2 shows that the range of values for the 109 determinations of iron (certified) is no greater than the range of 41 determinations of aluminum (not certified). For each element the three highest and three lowest values reported are shown together with the certified or mean value. What is critical to note is that uncertainty statements provided in the literature may be unfoundedly optimistic but are substantially improved when a certified value is available.

The situation is similar for lower concentrations, as shown in table 3. Here uncertainty estimates for titanium are in error by as much as 5000%. Note from tables 1 and 3 that at least six of the seven analysts reporting titanium do not include, within their uncertainties, the mean of the seven determinations.

## 4. Concern that SRMs Are Underutilized

It is very difficult to estimate the degree to which SRMs are utilized when their use is warranted. Review of sales records gives the rather imprecise “feeling” that usage in mining and manufacturing may exceed 10% of the applicable occasions. However, in the service sector, a 1% usage rate may be a better estimate. An open question is, “Are chemists receiving an adequate education in the use of Standard Reference Materials?”

In the academic and R&D sector a very rough idea of the rate of usage can be established by examining papers where the use of SRMs would have been helpful to validate new work. In tables 4 and 5, we have a survey of SRM use taken from periodicals spaced 10 years apart.

The precision of the data probably does not warrant concluding that usage rates have changed between 1977 and 1987. However, the data do provide gratifying evidence that researchers realize some value in reporting validation of new work by SRMs. I feel that a usage factor of at least 50% should be obtainable in this sector. Could it be obtained if reviewers of papers were a bit more insistent that reported results include SRMs when available?

## 5. Certification of SRMs

At NBS, we cannot rely only on assessments of measurement precision to set uncertainty limits [1]. Instead, an individually tailored program is set up for the project design, measurement, and certification of each SRM. Typically, this program will include measurement by more than one method and in more than one laboratory. Unless SRM units are individually certified, the program also must assess the homogeneity of the lot of material.

At each step of the program careful attention is given to precision of results from each method and each laboratory. Statistical assessments are made of homogeneity, which can be considered the material component to imprecision, in contrast to the precision of various measurements made on the material.

By the end of the measurement process, the measurement experts and the project manager have in hand far too much data to put onto a certificate. Their job is to distill those data into one meaningful uncertainty statement for each value certified. Obviously, they must take into account the precision of all the measurements and the homogeneity of the material, but, more importantly, they must zero in on the true value. They must crosscornpare data from different, independent methods. They often need to evaluate data from different laboratories. They must probe for systematic errors or bias in methods and instruments, including examination of such questions as recoveries and results on control samples.

Finally, they decide on the best possible description of the stated level of accuracy for the certification. Sometimes this parameter is stated as a tolerance interval; more often, it is simply given as an estimated uncertainty. The important factor to reiterate is that the uncertainty statement is more than a precision statement. Typically, the uncertainty will include the combined effects of method imprecision, possible systematic errors among methods, and material variability.

In cases where certification is for an element near the detection limit of state-of-the-art methods, it is usual for relative uncertainties to be large. For example, an element that can be certified at 1 ± 0.8 ng/g may seem to have an unacceptably high relative uncertainty of ±80%. However, the absolute error is quite small and defines the presence of one more element in the SRM to within ±0.8 ng/g. Until the methods for that particular element are improved, a certification with large relative uncertainty is all that is possible.

## 6. Consideration for SRM Users

As instrumental methods become very precise, users of a single, precise method begin to question why that method is so much more precise than the uncertainties on NBS certificates [1]. They wonder why NBS is “working at such low precision.” NBS uncertainty limits will always be wider than the precision obtained in any of the individual measurement methods used in certification.

It is good for users to have highly precise methods because they serve as a precursor to their attaining accurate measurement. In a sense it means they are ready to make full and effective use of SRMs to calibrate or validate their measurements. But users must be cautioned never to assume that good precision implies accuracy, without the confirmation by SRMs.

## Figures and Tables

**Table 1:** 1571 Orchard Leaves (μg/g)

Element	Literature [2]		Range
	NBS	*X* + *S*(*n*)	
Fe	300 ± 20	284 ± 28(109)	121–884
A1		320 ± 110 (41)	99–824
Sr	37 ± 1	37 ± 4 (39)	14.5–118
Ti		24 ± 9 (7)	2.4–191

**Table 2:** 1571 Orchard Leaves (μg/g) literature [2]

Element:	Iron	Aluminum
High:	884	824 ± 50
	450 ± 70	520 ± 180
	370 ± 45	460 ± 7
	300 ± 20^a^	320 ± 110^b^
Low:	205 ± 37	146 ± 20
	145 ± 4	140 ± 8
	121	103 ± 22

a Certified concentration.

b Literature mean for aluminum.

**Table 3:** 1571 Orchard Leaves (μg/g) literature [2]

Element:	Strontium	Titanium
High:	118	191 ± 33
	53 ± 4	96 ± 12
	45 ± 2	60
	37 ± 1^a^	24 ± 9^b^
Low:	31 ± 3.3	17.2 ± 0.3
	28.0 ± 0.6	6.6 ± 0.5
	14.5 ± 2.5	2.4 ± 0.4

a Certified concentration.

b Literature mean for titanium.

**Table 4:** Survey of three August 1977 periodicals to compare potential and actual use of SRMs

Periodical	Articles employing SRMs	
	Should have	Did
American Laboratory	Not available	
Applied Spectroscopy	4	0
Analytical Chemistry	10	4
Total	14	4
% Using	29%	

**Table 5:** Survey of three August 1987 periodicals to compare potential and actual use of SRMs

Periodical	Articles employing SRMs	
	Should have	Did
American Laboratory	7	4
Applied Spectroscopy	6	2
Analytical Chemistry	7	1
Total	20	7
% Using	35%	

## References

[1] Rasberry SD (1987). Reference Materials. Amer Lab.

[2] Gladney ES, Burns CE, Perrin DR, Roelandts I, Gills TE (1984). 1982 Compilation of Elemental Concentration Data for NBS Biological, Geological, and Environmental Standard Reference Materials.

